# Cell-free protein crystallization for nanocrystal structure determination

**DOI:** 10.1038/s41598-022-19681-9

**Published:** 2022-10-03

**Authors:** Satoshi Abe, Junko Tanaka, Mariko Kojima, Shuji Kanamaru, Kunio Hirata, Keitaro Yamashita, Ayako Kobayashi, Takafumi Ueno

**Affiliations:** 1 School of Life Science and Technology, Tokyo Institute of Technology, Nagatsuta-cho 4259, Midori-ku, Yokohama, 226-8501 Japan; 2 SR Life Science Instrumentation Unit, RIKEN/SPring-8 Center, 1-1-1, Kouto, Sayo-cho, Sayo-gun, Hyogo, 679-5148 Japan; 3 International Research Frontiers Initiative (IRFI), Tokyo Institute of Technology, Nagatsuta-cho 4259, Midori-ku, Yokohama, 226-8501 Japan; 4 Present Address: MRC Laboratory of Molecular Biology, Francis Crick Avenue, Cambridge, CB2 0QH UK

**Keywords:** Crystal engineering, Synthetic biology, X-ray crystallography, Nanocrystallography

## Abstract

In-cell protein crystallization (ICPC) has been investigated as a technique to support the advancement of structural biology because it does not require protein purification and a complicated crystallization process. However, only a few protein structures have been reported because these crystals formed incidentally in living cells and are insufficient in size and quality for structure analysis. Here, we have developed a cell-free protein crystallization (CFPC) method, which involves direct protein crystallization using cell-free protein synthesis. We have succeeded in crystallization and structure determination of nano-sized polyhedra crystal (PhC) at a high resolution of 1.80 Å. Furthermore, nanocrystals were synthesized at a reaction scale of only 20 μL using the dialysis method, enabling structural analysis at a resolution of 1.95 Å. To further demonstrate the potential of CFPC, we attempted to determine the structure of crystalline inclusion protein A (CipA), whose structure had not yet been determined. We added chemical reagents as a twinning inhibitor to the CFPC solution, which enabled us to determine the structure of CipA at 2.11 Å resolution. This technology greatly expands the high-throughput structure determination method of unstable, low-yield, fusion, and substrate-biding proteins that have been difficult to analyze with conventional methods.

## Introduction

Proteins crystallized in living cells have been frequently reported over the last few decades^1–3^. Such crystals provide biological functions such as protein storage, protection, heterogeneous catalysis, and immune system activation^4^. The relationships between the functions and structures of the crystals have been investigated by direct structure determination of the micron-sized crystals grown in living cells since the structure of polyhedra, a natural in-cell crystal, was first determined in 2007^5^. Thus, in-cell crystallization of various proteins has been widely expected to be developed as a next-generation structural biology tool because it does not require purification procedures and large-scale crystallization screening to obtain high-quality crystals^2^. In 2013, the crystal structure of cathepsin B from *Trypanosoma brucei* was determined using in-cell protein crystallization (ICPC) as the first example of determining the crystal structure of a recombinant protein^6^. Since then, ICPC has been attempted numerous times but structures of only a few proteins have been reported^4,7–11^. This is because crystals are often incidentally formed in cells and their size and quality are insufficient for structural analysis^12^. Therefore, several technical issues must be overcome in applying this method for protein structure analysis.

Several ICPC methods, such as high throughput screening and optimization of cell culture processes have been developed in efforts to resolve these problems^13–15^. A pipeline containing protein crystallization using insect cells with sorting by flow cytometry has been developed^15^. LaBaer's group constructed a set of baculovirus expression vectors for a large-scale parallel expression of proteins in insect cells and successfully prepared microcrystals^14^. Mammalian and insect cells, currently used for ICPC, continue to represent a significant limitation as platforms to produce large numbers of high-quality microcrystals rapidly. Although another attempt has been made to add chemical reagents used for in vitro crystallization, it has not led to improvements in ICPC, possibly because the efficiency with which the reagents penetrate cell membranes and their effects on other cellular functions are unknown^12^. *Bacillus thuringiensis* (Bt) bacteria have been used to express cry protoxins as crystallization vessels for cargo proteins recombinantly, but the structures could not be determined^16,17^. Once a new ICPC has been established and integrated with in vitro crystallization methods to overcome these concerns about ICPC, this method is expected to become a more accessible technology for structure analysis.

**Figure 1:**
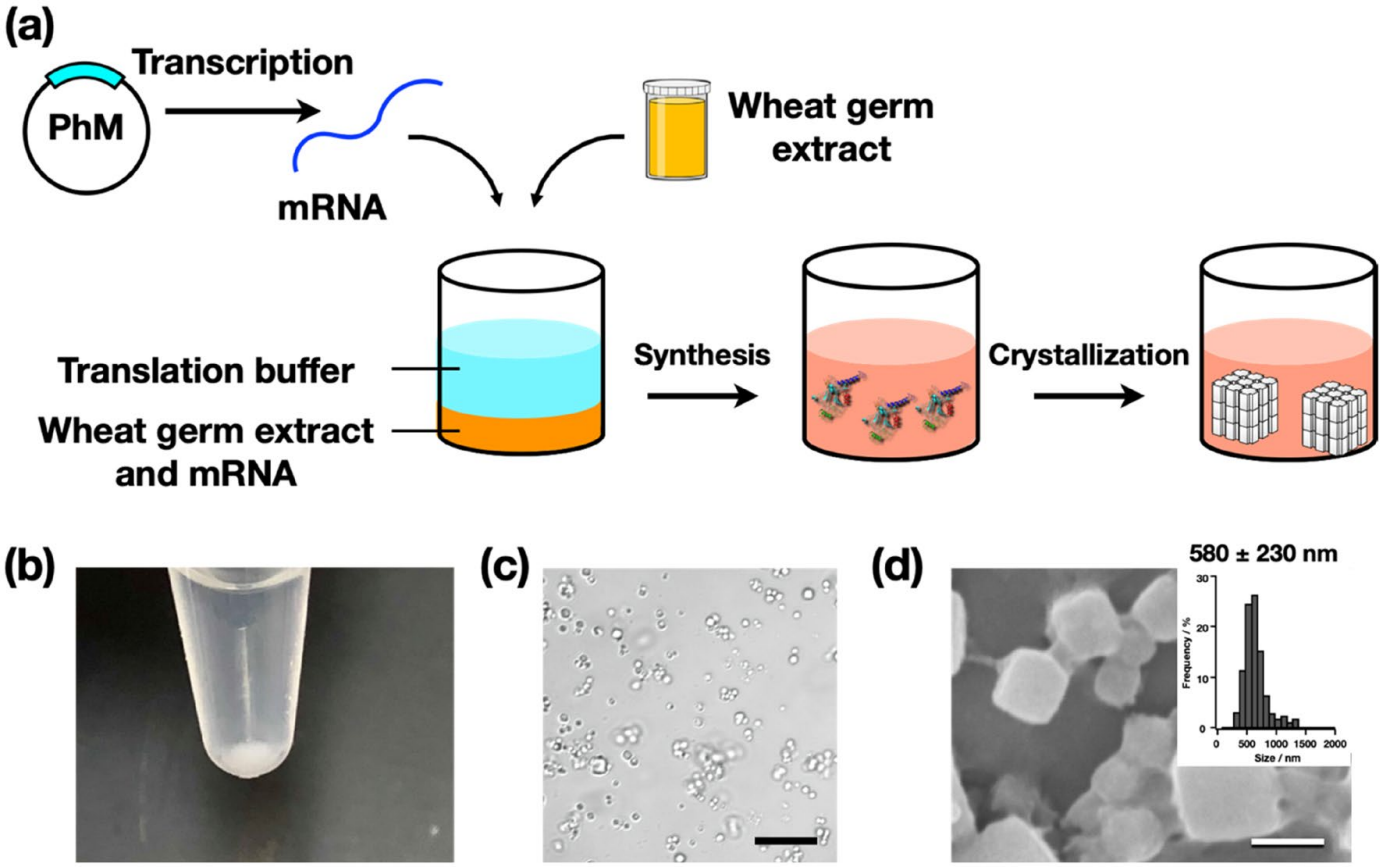
(**a**) Schematic illustration of cell-free protein crystallization (CFPC) of polyhedrin monomer (PhM) using the Wheat Germ Protein Synthesis kit. (**b**) Photograph of the tube after CFPC. (**c**) Differential interference contrast (DIC) image of **PhC_CF**. Scale bar 10 μm. (**d**) A scanning electron micrograph of **PhC_CF**. Size distribution of **PhC_CF** determined by the SEM image. Scale bar 1 μm.

Cell-free protein synthesis (CFPS), a protein preparation technique used in synthetic biology, is very effective for rapid screening of protein synthesis^18^. However, it has been considered unsuitable for structural biology efforts that require large amounts of protein, such as crystallization^19–21^. Here, we report the application of CFPS to ICPC (Fig. 1). We focus on (1) establishing crystallization of proteins using CFPS with small scale and rapid reactions and (2) manipulating the crystallization by adding chemical reagents. The polyhedra crystal (PhC) produced in insect cells by infection of cypovirus (cytoplasmic polyhedrosis virus, CPV) is one of the most highly studied in-cell protein crystals. We obtained nano-sized PhCs in a 200 μL reaction within 6 h. We determined the protein structure at high resolution using a current standard beamline (BL32XU) at SPring-8, a large synchrotron facility. PhC crystallized in a 20 μL reaction scale was successfully analyzed at 1.95 Å.

The most crucial advantage of CFPC is that various reagents can be added to the reaction mixture without preventing protein synthesis. The structure of crystalline inclusion protein A (CipA), a bacterial in-cell crystal, had not been previously reported^22^. Since we found that twin crystals are formed when CipA is expressed in *E. coli*, we applied CFPC to CipA with the addition of twin crystal inhibitors during the crystallization process and succeeded in obtaining suitable crystals and determining the structure of CipA at a 2.11 Å resolution. Therefore, CFPC, a hybrid method of ICPC and in vitro protein crystallization, can be developed at a surprisingly small scale to provide rapid crystallization without any purification procedures. CFPC opens up a new method for crystallizing unstable proteins and rapidly determining their structures.

## Results

### Crystallization of PhC by CFPC method

CFPS is a conventional synthetic biology method for protein structural determination^21^. It enables synthesis of proteins, such as membrane proteins and protein assemblies, that are difficult to purify using living cells^23,24^. We performed CFPS using extracts from wheat germ because these extracts have been identified as having the highest protein expression activity among the eukaryotic systems^25^. Crystallization of polyhedrin monomer (PhM) was performed using the Wheat Germ Protein Synthesis kit (WEPRO®7240 Expression Kit). The translation reaction was carried out using the bilayer method. A 20 μL reaction mixture containing 10 μL of WEPRO®7240 and 10 μL of the mRNA solution was overlaid with a 200 μL SUB-AMIX® SGC solution in a 1.5 mL microtube and then incubated at 20 °C for 24 h. White precipitates were collected after centrifuging the reaction solution (Fig. 1b). The crystalline precipitate was observed with an optical microscope (Fig. 1c). SDS-PAGE and matrix assisted laser desorption ionization-time of flight mass spectrometry (MALDI-TOF MS) of the precipitate showed a band at 28 kDa and a peak of 28,361 Da, respectively (Supplementary Fig. 1). These results are consistent with the calculated molecular weight of the PhM (28,368 Da). The crystals prepared from the CFPC reaction (**PhC_CF**) have the same cubic morphology as that of PhC synthesized in insect cells (PhC_IC). The average size of **PhC_CF** (580 nm) is approximately one-fifth of that of PhC_IC (2700 nm) as determined by scanning electron microscopy (SEM) (Fig. 1d, Supplementary Fig. 2).

### Time course and temperature dependency of the PhC_CF formation

**Figure 2:**
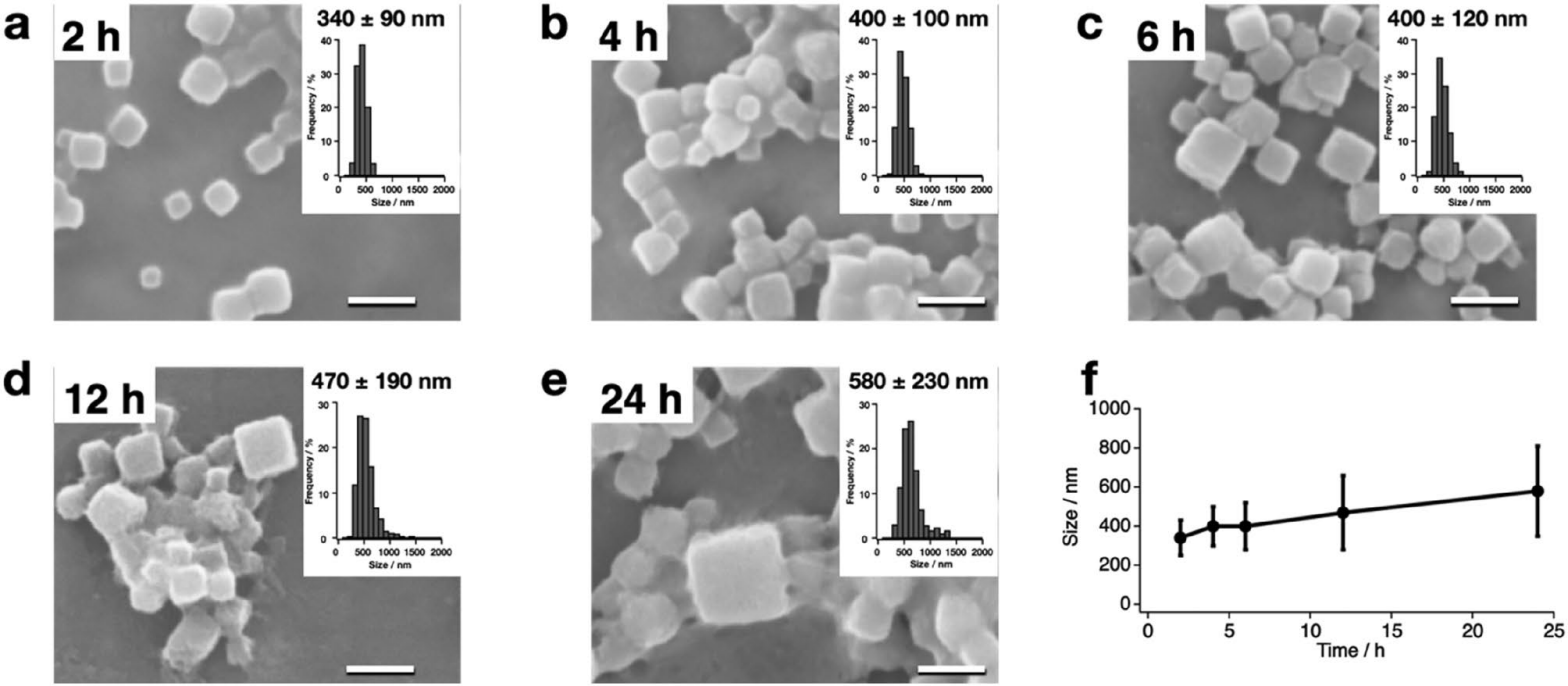
Time-dependent CFPC of **PhC_CF**. SEM images and size histograms of the purified **PhC_CF** after translation at 20 °C for (**a**) 2 h, (**b**) 4 h, (**c**) 6 h, (**d**) 12 h, and (**e**) 24 h. (**f**) Crystal size of purified **PhC_CF** over time. Error bars = SD. Scale bars 1 μm.

To clarify the time dependency of the **PhC_CF** formation, the crystallization reaction induced by CFPC was monitored at 0.5 h, 1 h, 1.5 h, 2 h, 4 h, 6 h, 12 h, and 24 h with SEM. When PhM was expressed at 20 °C, the cubic crystals were first observed 2 h after the initiation of the translation reaction (Fig. 2). The average sizes of the crystals measured after 2 h, 4 h, 6 h, 12 h, and 24 h were found to be 340 nm, 400 nm, 400 nm, 470 nm, and 580 nm, respectively. There were no crystals observed by SEM after 1.5 h of the reaction. When the expression of PhM was confirmed by SDS-PAGE at 0.5 h, 1 h, 1.5 h, 2.0 h, 3.0 h, and 4.0 h after the translation reaction at 20 °C, a band corresponding to PhM was observed after 1 h, but the band was not observed at 0.5 h (Supplementary Fig. 3). These results indicate that insufficient amounts of PhM for the crystallization are obtained after 1.5 h.

**Figure 3:**
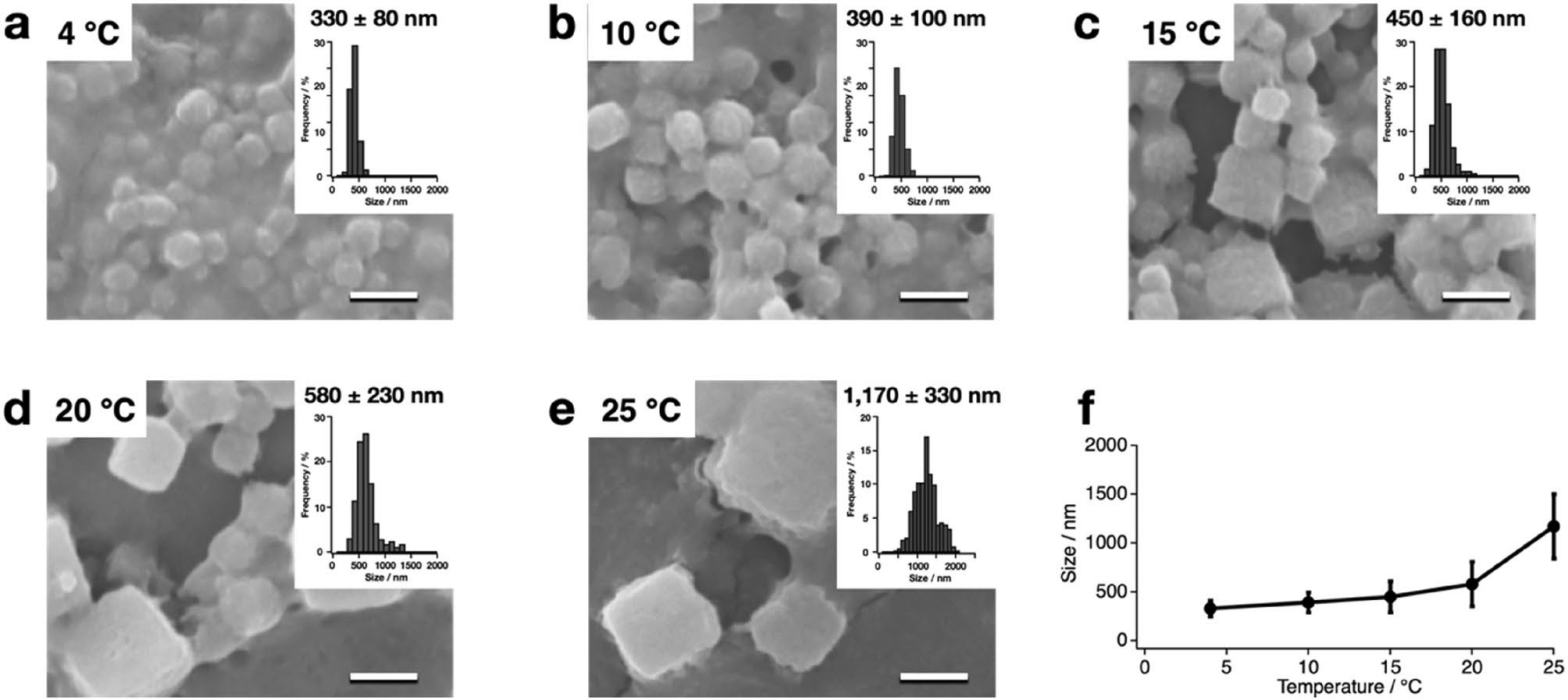
Temperature-dependent CFPC of **PhC_CF**. SEM images and size histograms of the purified **PhC_CF** after translation at (**a**) 4 °C, (**b**) 10 °C, (**c**) 15 °C, (**d**) 20 °C, and (**e**) 25 °C for 24 h. (**f**) Crystal size of purified **PhC_CF**s over temperature. Error bars = SD. Scale bars 1 μm.

To evaluate the temperature dependency of the **PhC_CF** formation, the translation reactions were performed at various temperatures. After 24 h, the crystals were formed with average sizes of 330 nm, 390 nm, 450 nm, 580 nm, and 1170 nm at 4 °C, 10 °C, 15 °C, 20 °C, and 25 °C, respectively (Fig. 3). At 15 °C and 25 °C, cubic crystals were observed, but many round crystals were observed at 4 °C and 10 °C. Although the reaction temperature is expected to affect the expression efficiency^19^, the yields of PhM after 24 h did not differ significantly in the temperature range of 10–20 °C, as confirmed by SDS-PAGE (Supplementary Fig. 4). SDS-PAGE of the reaction solution after CFPC, the supernatant solution after centrifugation, and purified crystals to compare the amounts of PhM in PhC and remaining in the solution showed that approximately 70–80% of PhMs form PhCs at 10–20 °C, but the yields were lower at 4 and 25 °C (Supplementary Fig. 4). Therefore, the crystal size and morphology are expected to be affected by the amount of crystallized PhM and the crystallization rate at various temperatures.

### Structural analysis of the nano-sized PhC_CFs

**Figure 4:**
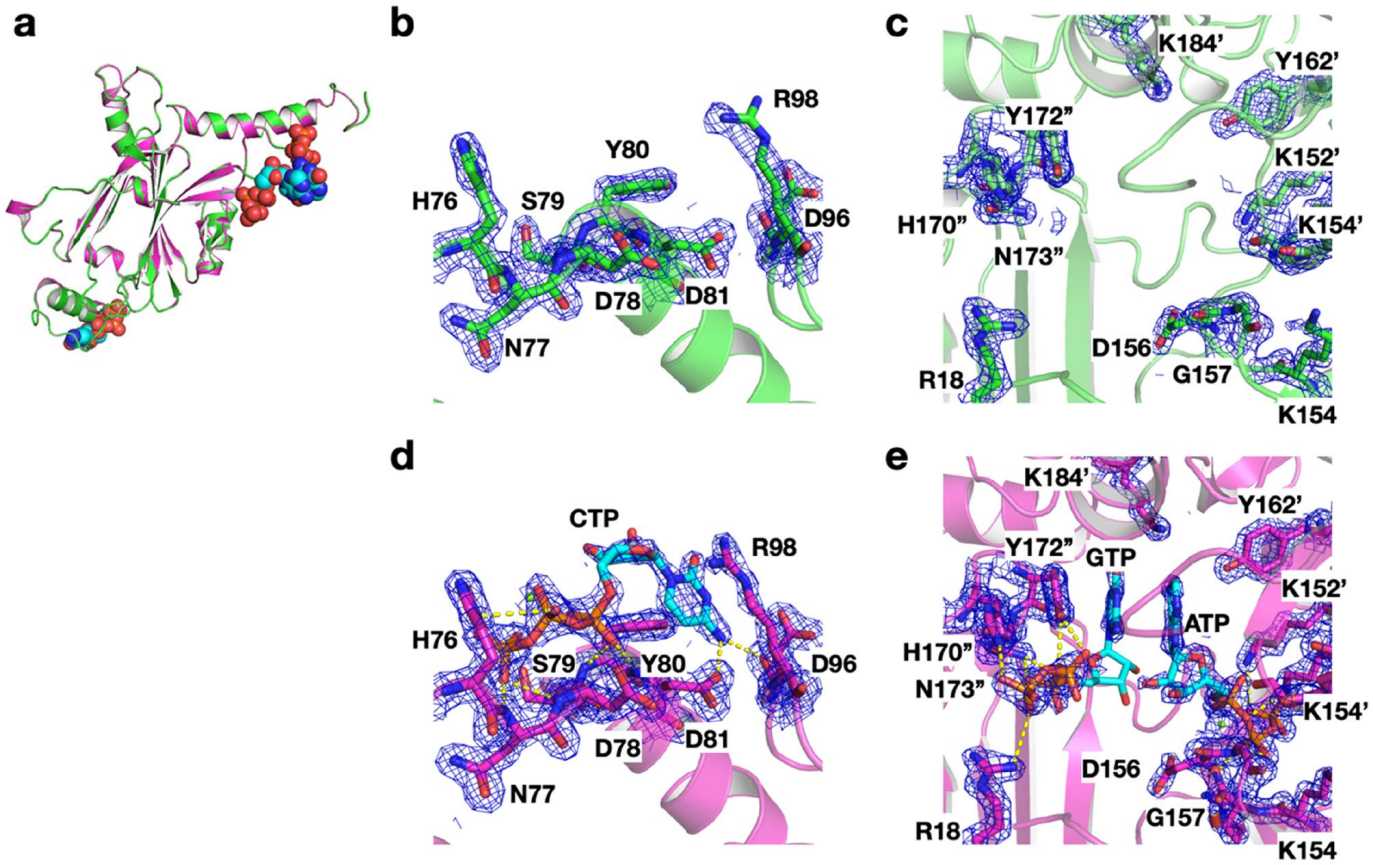
Comparison of crystal structure between **PhC_CF**_**20°C/24h**_ and PhC_IC (PDB ID: 5gqm)^28^. (**a**) Superimposed structures of **PhC_CF**_**20°C/24h**_ (green) and PhC_IC (magenta). (**b**,**c**) Close-up views of (**b**) CTP and (**c**) ATP/GTP binding sites in **PhC_CF**_**20°C/24h**_, (**d**,**e**) Close-up views of (**d**) CTP and (**e**) ATP/GTP binding sites in PhC_IC. The selected 2|*F*_o_| − |*F*_c_| electron-density maps at 1.0σ are shown in bule. Hydrogen bonds are indicated with yellow dotted lines.

To collect diffraction data from the nano-sized **PhC_CFs**, the crystals isolated from the reaction mixture were diffracted using the micro-X-ray beam of the BL32XU beamline equipped with Serial Synchrotron Rotation Crystallography (SS-ROX) at SPring-8 (Supplementary Fig. 5)^26,27^. **PhC_CF** obtained after 24 h at 20 °C (**PhC_CF**_**20°C/24h**_) was refined with a resolution of 1.80 Å, and has a space group (*I*23) and lattice parameters which are essentially identical to those of PhC_IC (PDB ID: 5gqm) (Tables 1, 2). The root mean square deviation (RMSD) value of the Cα atoms for the structure from PhC_IC is 0.09 Å (Fig. 4a). The main difference between **PhC_CF**_**20°C/24h**_ and PhC_IC is that **PhC_CF**_**20°C/24h**_ shows no electron density of nucleotide triphosphates (NTPs) bound to the monomer interface, which are observed in PhC_IC (Fig. 4b–e). The average *B*-factor values per residues of all atoms in **PhC_CF**_**20°C/24h**_ show a large value for His76 because of the lack of cytosine triphosphate (CTP) interacting with His76 in PhC_IC (Fig. 4b,d, Supplementary Fig. 6). In PhC_IC, the amino acid residues surrounding guanine triphosphate (GTP) and adenosine triphosphate (ATP) interact with the NTPs, but in **PhC_CF**_**20°C/24h**_, which lacks these electron densities, there is no significant difference in the side chain conformation between **PhC_CF**_**20°C/24h**_ and PhC_IC (Fig. 4b–e). These results indicate that the NTP binding is not essential for the crystallization of PhM.

**Table 1:** Crystal size and Crystallographic data of **PhC_CF**.

Crystals	Temperature (°C)	Reaction Time (h)	Crystal size (nm)	Resolution (Å)
**PhC_CF**_**20°C/1h**_	20	1	–^*a*^	–^*b*^
**PhC_CF**_**20°C/2h**_	20	2	340 ± 90	–^*b*^
**PhC_CF**_**20°C/4h**_	20	4	400 ± 100	–^*b*^
**PhC_CF**_**20°C/6h**_	20	6	400 ± 120	2.50
**PhC_CF**_**20°C/12h**_	20	12	470 ± 190	2.18
**PhC_CF**_**20°C/24h**_	20	24	580 ± 230	1.80
**PhC_CF**_**4°C/24h**_	4	24	330 ± 80	–^*b*^
**PhC_CF**_**10°C/24h**_	10	24	390 ± 100	–^*b*^
**PhC_CF**_**15°C/24h**_	15	24	450 ± 160	2.20
**PhC_CF**_**25°C/24h**_	25	24	1170 ± 330	1.87
**PhC_CF**_**20μL_20°C/24h**_	20	24	610 ± 150	1.95
PhC_IC	27	72	2710 ± 870^*c*^	1.70^*d*^

^a^No crystals observed by SEM. ^b^No data sets were obtained. ^c^Supplementary Fig. 2. ^d^Reference^28^.

**Table 2:** Crystallographic data of **PhC_CF**.

	20 °C/2 h	20 °C/4 h	20 °C/6 h	20 °C/12 h	20 °C/24 h	4 °C/24 h	10 °C/24 h	15 °C/24 h	25 °C/24 h	20μL_ 20 °C/24 h
No. of loops	4	4	4	4	4	4	4	4	4	4
No. of indexed images	1	91	1531	8754	12,846	0	258	3834	3807	12,355
Space group			*I*23	*I*23	*I*23			*I*23	*I2*3	*I2*3
Unit cell (Å) *a* = *b* = *c*			104.3	103.3	104.4			104.4	103.7	103.6
Resolution range (Å)			50–2.50 (2.51–2.50)	50–2.18 (2.19–2.18)	50–1.80 (1.81–1.80)			50–2.20 (2.21–2.20)	50–1.87 (1.88–1.87)	50–1.95 (1.96–1.95)
Observed reflections			427,843 (6586)	343,3242 (59,651)	6,907,468 (114,912)			1,489,024 (27,270)	2,048,341 (33,158)	6.106,562 (95,158)
Unique reflections			6679 (148)	9832 (253)	17,297 (414)			9786 (261)	15,459 (367)	13,641 (309)
Completeness (%)			100 (100)	100 (100)	100 (100)			100 (100)	100 (100)	100 (100)
Multiplicity			64.1 (44.5)	349 (236)	399 (277)			152.2 (105)	133 (90)	447.7 (308)
I/σ			3.8 (1.24)	7.3 (1.28)	5.5 (1.53)			4.8 (1.14)	4.5 (1.33)	7.3 (1.23)
CC_1/2_			0.9457 (0.5054)	0.98821 (0.5854)	0.9899 (0.5326)			0.9759 (0.4000)	0.9696 (0.4921)	0.9908 (0.6707)

Values in parentheses are for the highest-resolution shell.

The crystals formed with CFPC under various conditions provided data sets suitable for structural analysis (Tables 1, 2, 3). The crystal structures were refined with a resolution range of 1.80–2.50 Å. The RMSD values of the Cα atoms from PhC_IC are less than 0.29 Å, indicating that the overall structure of PhC_IC is retained in **PhC_CFs**. While no data sets were obtained for the crystals formed at 20 °C for 2 h and 4 h, and at 4 °C and 10 °C for 24 h due to fewer indexed images, we obtained a data set for **PhC_CF**_**20°C/6h**_ with a resolution of 2.50 Å (Tables 1, 2, 3). The resolution was improved when the reaction time was increased from 6 to 24 h. This improvement in resolution is probably attributed to an increase in the indexed images due to the larger crystal size (Fig. 2f). These results show that the CFPC reaction of PhC under optimized conditions successfully produces nanocrystals with sufficient quality to obtain a high-resolution structure in only 6 h. This reaction time is dramatically reduced from the cultivation time (> 3 days) required to obtain comparable high-quality crystals using insect cells^15^.

**Table 3:** Refinement statics of **PhC_CF**.

	20 °C/2 h	20 °C/4 h	20 °C/6 h	20 °C/12 h	20 °C/24 h	4 °C/24 h	10 °C/24 h	15 °C/24 h	25 °C/24 h	20μL_ 20 °C/24 h
Resolution range (Å)	–	–	42.58–2.50	36.63–2.18	42.30–1.80	–	–	36.9–2.20	42.31–1.87	36.63–1.95
Reflection used			6679	9829	17,297			9785	15,457	13,640
R-factor (%)			17.36	17.17	15.44			17.27	17.16	15.81
Free R-factor (%)			26.79	23.53	18.66			24.07	22.56	19.94
**R.m.s.deviations from ideal**										
Bond length (Å)			0.008	0.008	0.014			0.008	0.010	0.014
Angle (°)			1.12	0.96	1.77			0.95	1.06	1.72
**Ramachandran plot (%)**										
Favored region			96.73	97.96	97.96			96.73	97.55	97.55
Allowed region			3.27	2.04	2.04			3.27	2.45	2.45
Outlier region			0.0	0.0	0.0			0.0	0.0	0.0

### Validation of CFPC using minimum reaction volume

One of the most critical features of CFPC is the minimization of the reaction scale. The reaction was carried out by dialyzing a mixture of WEPRO®7240 (5 μL), the mRNA solution (5 μL) and 10 μL SUB-AMIX® SGC against 1.0 mL SUB-AMIX® SGC. After 24 h at 20 °C, crystals (**PhC_CF**_**20μL_20°C/24h**_) were collected. The average size of **PhC_CF**_**20μL_20°C/24h**_ was 610 ± 150 nm, which is essentially identical to the average size of **PhC_CF**_**20°C/24h**_ (Supplementary Fig. 7). The structural analysis of **PhC_CF**_**20μL_20°C/24h**_ obtained with a 20 μL reaction scale by SS-ROX gave a data set of 1.95 Å resolution, which is comparable to those of the PhC obtained with a 220 μL scale (Tables 1, 2, 3).

### Structure determination of CipA by CFPC

After successfully validating CFPC in crystallizing PhC, we applied this method to overcome current challenges in structure determination of CipA since it provides an opportunity to add chemical compounds during crystallization. Crystalline inclusion protein A (CipA), a hydrophobic protein of 104 amino acid residues, spontaneously forms crystalline aggregates in *Photorhabdus luminescens*, an entomopathogenic bacterium^29^. Its native function is postulated to be involved in nematode symbiosis or pathogenesis^30^. It can also form a crystallized aggregation in *E.coli*, which is used as a template for constructing solid-nanomaterials. Its structure has not yet been determined^22^. CipA crystals formed in *E. coli* (CipAC_EC) with an average size of 410 ± 80 nm were diffracted with a resolution of 2.8 Å using the SS-ROX method at the BL32XU beamline of SPring-8 (Supplementary Fig. 8a,b, Tables 4, 5). However, we could not find MR solution when using the predicted AlphaFold2 (AF2) model^31^. This is because of the large twin fraction of 0.42 (Table 4). Next, we attempted to determine the structure of CipA by reducing the twin fraction using CFPC. When CipA was expressed by CFPC with dialysis method^32^, a white precipitate appeared in the solution mixture after 24 h (Supplementary Fig. 8c). The SEM image and the MALDI TOF–MS of the precipitate showed that the precipitate is the CipA crystal (**CipAC_CF**) with an average size of 3400 ± 880 nm (Supplementary Fig. 8d,e). This is eight times larger than the size of CipAC_EC. Structural analysis of **CipAC_CF** was attempted with data of a resolution of 1.61 Å obtained using the small wedge method at BL32XU of SPring-8^27^. However, the structure could not be determined because the twin fraction was still too high (0.42), as it is in *E.coli*. To overcome the twinning issue occurring in the high quality crystals, we recognized that the CFPC method permits addition of reagents which inhibit twinning, such as ethanol, 1,4-dioxane, PEGs, Dextran, and TEG^33,34^. The SEM images show that the **CipAC_CFs** crystallized in the presence of additives have slightly larger or similar sizes and similar shapes relative to **CipAC_CF** crystallized in the absence of additives (Supplementary Fig. 9). This indicates that the CFPC method can be expanded to include various chemical manipulations to crystals, as well as addition of chemical compounds and proteins to crystals and proteins during the crystal growth process.

**Table 4:** Crystallographic data of CipAC_EC and **CipAC_CF.**

	CipAC_EC	**CipAC_CF**							
		No additive	EtOH	Dioxane	PEG400	PEG3350	PEG8000	Dextran	TEG
			3 v/v%	3 v/v%	1 v/v %	1 w/v %	1 w/v %	2 w/v%	1 v/v %
Space group	*I*4	*I*4	*I*4	*I*4	*I*4	*I*4	*I*4	*I*4	*I*4
Unit cell (Å) *a* = *b*, and *c*	61.2, 53.8	60.4, 53.2	61.1, 54.0	61.1, 54.0	61.3, 54.2	61.1, 54.0	61.1, 54.0	61.0, 54.0	61.2, 54.1
Resolution range (Å)	50–2.80 (2.81–2.80)	50–1.61 (1.71–1.61)	50–1.98 (2.10–1.98)	50–2.11 (2.24–2.11)	50–2.47 (2.62–2.47)	50–2.38 (2.52–2.38)	50–2.08 (2.21–2.08)	50–1.98 (2.10–1.98)	50–2.48 (2.63–2.48)
Observed reflections	94,065 (1261)	3,412,217 (480,752)	338,736 (50,925)	111,712 (18,248)	46,092 (7,428)	43,814 (7,064)	253,733 (37,516)	338,784 (52,079)	68,170 (11,178)
Unique reflections	2504 (62)	12,425 (2025)	6991 (1125)	5787 (935)	3662 (582)	4080 (641)	6050 (994)	6987 (1140)	3595 (574)
Completeness (%)	100 (100)	100 (100)	100 (100)	100 (100)	99.9 (100)	99.9 (100)	100 (100)	100 (100)	100 (100)
Multiplicity	37.6 (20.3)	274.5 (237.4)	48.5 (45.3)	19.3 (19.5)	12.6 (12.8)	10.7 (11.0)	41.9 (37.7)	48.5 (45.7)	19.0 (19.5)
I/σ	5.2 (1.2)	25.39 (0.7)	12.46 (1.6)	9.04 (1.7)	6.49 (1.5)	7.09 (1.8)	11.01 (1.6)	13.22 (1.7)	8.73 (1.6)
CC_1/2_	0.9139 (0.317)	0.999 (0.593)	0.991 (0.543)	0.994 (0.587)	0.979 (0.499)	0.979 (0.375)	0.993 (0.526)	0.992 (0.693)	0.980 (0.589)
< *I*^2^ > / < *I* >^2a^	1.565	1.537	1.584	1.893	1.954	1.751	1.741	1.592	1.732
Twin fraction^a^	0.42	0.42	0.38	0.10	0.14	0.28	0.22	0.36	0.30

^a^Twinning was analyzed with *phenix.xtriage*. Values in parentheses are for the highest-resolution shell.

**Table 5:** Refinement statics of CipAC_EC and **CipAC_CF**.

	CipAC_EC	**CipAC_CF**							
		No additive	EtOH	Dioxane	PEG400	PEG3350	PEG8000	Dextran	TEG
			3 v/v%	3 v/v%	1 v/v %	1 w/v %	1 w/v %	2 w/v%	1 v/v %
Resolution range (Å)	43.29–2.80	30.17–1.61	30.55–1.98	43.2–2.11	43.32–2.47	27.01–2.38	30.57–2.08	30.51–1.98	30.58–2.48
Reflection used	2504	12,417	6989	5781	3658	4076	6048	6985	3593
R-factor (%)	29.8	37.31	33.60	18.69	23.14	29.48	22.50	34.78	30.21
Free R-factor (%)	43.8	41.29	39.00	22.24	28.96	40.16	26.76	40.78	36.48
**R.m.s.deviations from ideal**									
Bond length (Å)	0.015	0.010	0.010	0.009	0.011	0.067	0.010	0.012	0.010
Angle (°)	1.99	1.50	1.40	1.16	1.64	3.25	1.29	1.38	1.45
**Ramachandran plot (%)**									
Favored region	70.73	94.12	91.18	97.75	88.10	94.37	96.55	94.03	98.33
Allowed region	19.51	5.88	8.82	2.25	9.52	5.63	3.25	5.97	1.67
Outlier region	9.76	0.00	0.00	0.00	2.38	0.00	0.00	0.00	0.00

**Figure 5:**
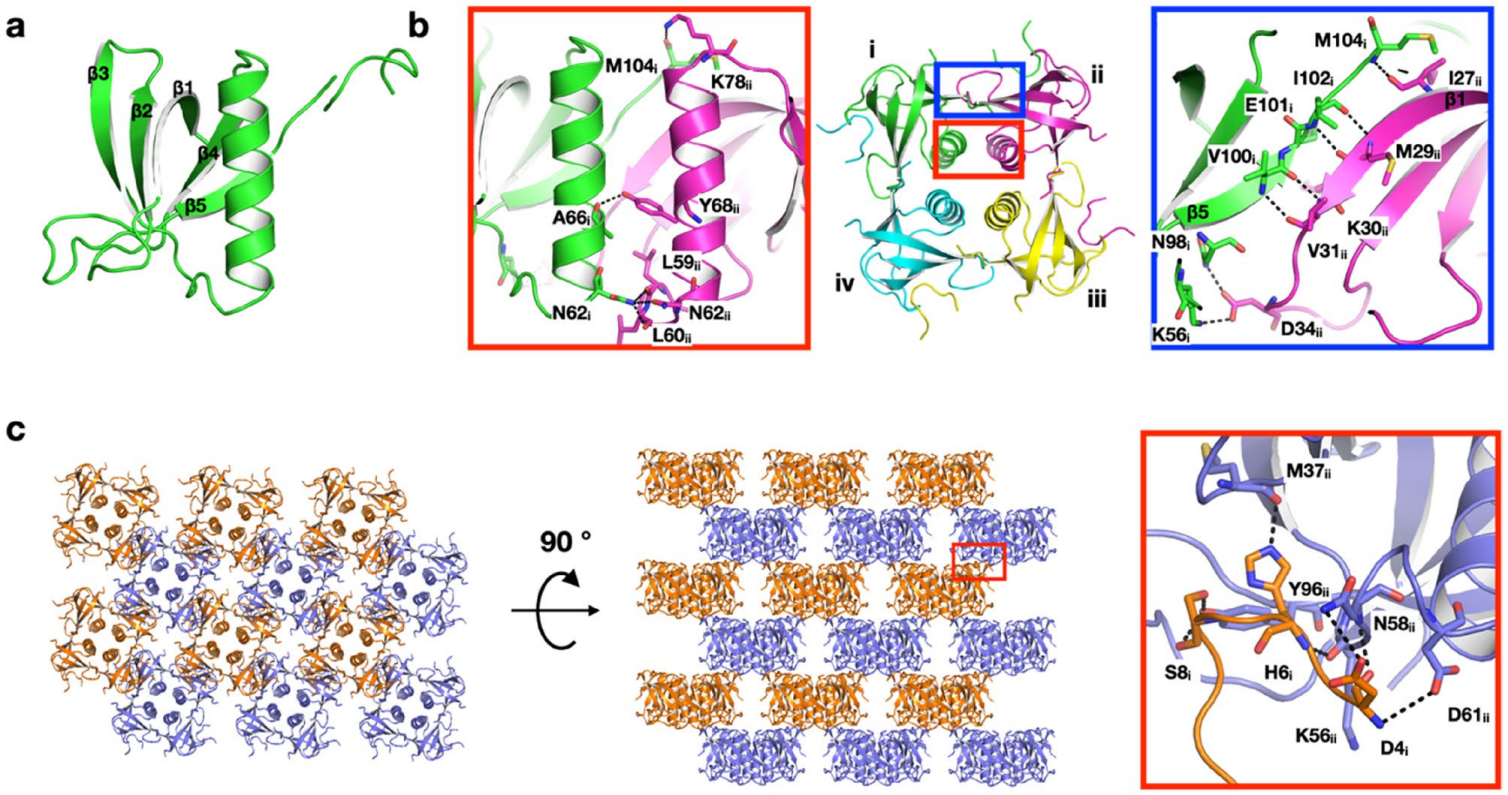
Crystal structure of **CipAC-CF** with 1,4-dioxane. The structure of (**a**) monomer and (**b**) tetramer. (**a**) CipA monomer are consists of the N-terminal arm followed by three β-strands β1, β2, β3, α-helix, and β-strands β4 and β5. (**b**) the interactions between monomers (i, ii, iii and iv) in the tetramer. (**c**) Lattice structure and interactions between tetramers. Hydrogen bonds are indicated with black dotted lines.

X-ray diffraction experiments of the crystals showed that **CipAC_CF** crystallized in the presence of 3 v/v % 1,4-dioxane dramatically reduces the twin fraction to 0.10, with a resolution of 2.11 Å (Table 4, 5). The crystal is tetragonal, with a *I*4 space group having unit cell parameters *a* = *b* = 60.1 Å, *c* = 54.0 Å, α = β = γ = 90° (Tables 4, 5). The structure was determined by the molecular replacement method using the search model created by AF2^31^ via ColabFold^35^. The structure of the CipA monomer consists of the N-terminal arm followed by three β-strands β1, β2, β3, α-helix, and β-strands β4 and β5. Except for the N-terminal arm, the globular domain is a typical oligonucleotide/oligosaccharide-binding (OB) fold (Fig. 5a). The structures of Met1-Asn3 and Asp12-Val19 at the N-terminus could not be determined due to the luck of electron density. In the crystal lattice, the four α-helices from each monomer form a four-helix bundle around the crystallographic fourfold symmetry axis and exist as a tetramer. This tetramer is consistent with the results of PISA^36^ prediction of oligomeric states and is considered the basic unit of crystal growth. As for the interactions between monomers in the tetramer, hydrogen bonds (N_δ_/Asn62_i_–O/Leu59_ii_, N_δ_/Asn62_i_–O/Leu60_ii_, N_δ_/Asn62_i_–O_δ_/Asn62_ii_, O/Ala66_i_–O_ζ_/Tyr68_ii_, and O/Met104_i_–N_ζ_/Lys78_ii_) are formed between each helix (Fig. 5b). In addition, the β1 and β5 strands of the neighboring molecule form a new β-sheet with hydrogen bonds (N_ζ_/Lys56_i_–O_δ_/Asp34_ii_, N_δ_/Asn98_i_–O_δ_/Asp34_ii_, N/Val100_i_–O/Val31_ii_, O/Val100_i_–N/Val31_ii_, N/Ile102_i_–O/Met29_ii_, N/Ile102_i_–N/Met29_ii_, and N/Met104_i_–O/Ile27_ii_) and salt bridge (N_ζ_/Lys56_i_–O_δ_/Asp34_ii_) (Fig. 5b). The interaction between the edges of each tetramer forms the basic crystal lattice, and it is further stabilized by the embedded N-terminal arm of the neighboring monomer molecule at the cleft created between the tetramer-tetramer interface (N/Asp4_i_-O_δ_Asp61_ii_, O/Asp4_i_-N/Asn58_ii_, O_δ_/Asp4_i_-N_δ_/Asn58_ii_, N/His6_i_-O/Lys56_ii_, N_ε_/His6_i_-O/Met37_ii_, O_γ_/Ser8_i_-O_ζ_/Tyr96_ii_, and O/Ser8_i_-O_ζ_/Tyr96_ii_) (Fig. 5c).

A search for similar structures on the Dali server^37^ shows OB-fold domain-containing proteins, as expected. Among them, a pentameric B subunit of heat-labile enterotoxin type IIB (PDB ID: 1QB5) from a pathogenic bacterium with high structural similarity (z-score > 9.0) was found. Even though the sequence homology was less than 10%, the topology of the monomers of this protein and CipA is very similar, with an RMSD value of 2.2 Å in equivalent Cα atoms. Although these proteins have different oligomerization states, each monomer forms a bundle of α-helices around the central symmetry axis, and the β-strands at adjacent monomers form β-sheets at the outer rim of the complex (Supplementary Fig. 10).

## Discussion

We have succeeded in determining crystal structures of two types of proteins with high-resolution using CFPC. ICPC of both PhC and CipA are believed to be influenced by the complex environment of living cells^5,22^. However, a comparison of the structures of PhCs by ICPC and CFPC suggests that the encapsulation of NTPs in PhCs is not essential for the crystallization. For the crystallization of CipA, the formation of the twin crystal by ICPC could be inhibited by the addition of foreign molecules in CFPC to obtain crystals used for the structural analysis. These results suggest that strict cellular environments are not required for their crystallization. Thus, by using CFPC, we were able to determine the crystallization factors in cell environments, which were difficult to investigate by ICPC alone. Protein crystallization using CFPC indicates that there are opportunities to obtain crystals of recombinant proteins because additives can control the crystallization, as with in vitro crystallization.

Protein crystals with high-resolution diffraction data were obtained from small reaction volumes and short CFPC timeframes. CFPC of PhC allowed us to obtain the 2.50 Å structure for crystals obtained only 6 h after initiating the reaction. ICPC using insect cells requires three days to obtain PhC_IC after virus infection (Table 1)^15^. This is because insect cells involve various cellular processes in producing the target protein. The CFPS reaction system is dedicated to producing the target protein and the crystals. Furthermore, CFPC using cell extracts allows crystal formation independent of the reaction scale. Using a small dialysis cup, not only can PhC_CF be identified at a reaction scale of only 20 μL, but a structure with a resolution of 1.95 Å was also obtained. Thus, it was found that CFPC can efficiently synthesize high-quality protein crystals by taking advantage of the smaller reaction scale. This provides a solution to the problem of low yields of high-quality crystals produced by ICPC and shows great potential to facilitate crystal screening, which is not feasible using previously reported methods and ICPC.

The crystal structure of CipA was determined at high resolution by adding chemical reagents to the CFPC reaction mixture. **CipAC_CF** forms a multilayer structure composed of the tetramers as the building blocks (Fig. 5). Between the layers, the N-terminal Asp4-Ser8 interacts with another layer to stabilize the crystals. The layered stacking of the tetramers forms porous space in the lattice structure. The region from Asp12-Val19, whose model structure has not been determined due to disorder, is expected to be located in that porous space.

No electron density of other molecules, including 1,4-dioxane used as an additive, was observed at the interface of CipA. In addition to 1,4-dioxane, which is known to be a twin inhibitor^38^, PEGs were found to reduce the twin fraction (Table 4). In particular, a significant reduction by PEG400 was observed. The effect is presumed to be similar to the effect provided by 2-methyl-2,4-pentanediol (MPD), rather than the exclusion volume effect of PEG with a large molecular weight^33^. On the other hand, CipAC_EC prepared by ICPC gave high-resolution diffraction data, but the structure could not be determined due to the high twin fraction (Table 4). In *E. coli*, a lack of interacting molecules, such as PEG400, on the surface of CipA may lead to incorporation of inverted tetramers into the multilayered crystal structure of CipA_EC, resulting in a large twin fraction (Supplementary Fig. 8). In this way, CFPC permits control of the crystallization process to obtain high-quality protein crystals by adding chemical reagents.

CFPC is useful for investigating crystallization steps in living cells. Several model proteins have been used to study the ICPC mechanism^12^. These model proteins can be crystallized even in an impurity-rich intracellular environment. These studies suggest that abundant intracellular and organelle endogenous proteins play roles as precipitants or crowding agents, like polyethylene glycols. Furthermore, ICPC has been attempted by adding a crystallization reagent, but no characteristic effect on the crystallization has been confirmed^12^. Since the intracellular reactions induced by ICPC are complex, it remains challenging to identify common crystallization factors for model proteins. In this study, the addition of PEG was found to inhibit twinning and promote the formation of high-quality crystals with a larger size (Supplementary Figs. 8, 9). It has been reported that PEG induces liquid–liquid phase separation (LLPS) in the reaction of CFPS^39^. Furthermore, LLPS plays an important role during in vitro protein crystallization^40^. Thus, it is inferred that the LLPS is one factor involved in promoting the crystallization of polyhedra and CipA in the CFPC solution. A detailed study of this proposal is underway.

## Conclusion

We established a CFPC method to rapidly obtain protein crystals in microliter volumes within a few hours without complicated purification and crystallization procedures. Furthermore, high-resolution structures of proteins were obtained using the nanocrystals. Although ICPC has been expected to become an important tool in crystal structure analysis, crucial challenges remain because the crystals are not formed in suitable amounts and quality to provide high-resolution structures. We used CFPC to enable rapid screening of reaction conditions such as temperature, time, and the effects of additives and achieved preparation of high-quality protein crystals suitable for structure analysis. These results indicate that CFPC will likely be a powerful tool for crystal structure analysis of unstable or low-yield proteins, which are currently considered challenging to crystallize using conventional protein crystallization methods.

## Methods

### Materials

All reagents were purchased from TCI, Wako, Nacalai Tesque, Sigma–Aldrich, and Life Technologies and were used without further purification.

### Scanning electron microscopy (SEM)

The morphologies of purified crystals were confirmed by scanning electron microscopy (SEM). After substituting PBS with Milli-Q water, the crystals were dried and observed by SEM. SEM analyses were performed on JCM-6000 Neoscope (JEOL). Crystals of WTPhC and CipA did not dissolve in Milli-Q water^5,30^.

### Cell-free synthesis and crystallization of PhM

Expression and crystallization of PhM were performed using a WEPRO7240 Expression Kit (CellFree Sciences). The gene of polyhedrin was cloned into the PEU-E01-MCS vector (CellFree Sciences) for the polyhedrin expression. The plasmid was amplified in DH5α bacteria and purified using the Qiagen Plasmid Midi Kit. Transcription was performed in 1.5 mL tubes according to the protocol of the expression kit. After incubation for 6 h at 37 °C, mRNA was used for translation. Translation reactions were carried out using the bilayer method. 20 μL of reaction mixture containing 10 μL of WEPRO7240 wheat germ extract, 10 μL of prepared mRNA, 40 mg/mL creatine kinase was overlaid with 200 μL of SUB-AMIX SGC solution in a microtube and incubated at 20 °C for 24 h. The crystals were collected by centrifuge and washed with PBS several times. Time-dependent and temperature-dependent crystallization were performed by the same method except for the change of time and temperature.

### X-ray crystal structure analysis of PhC_CFs

Before the data collection, the crystals were immersed in PBS buffer containing 50% ethylene glycol and then picked up with a 1000 μm Dual Thickness MicroLoops (Mitegen), followed by frozen in liquid nitrogen. The data diffraction of PhCs was collected at 100 K using the beamline of BL32XU at SPring-8 with an X-ray wavelength of 1.00 Å. The whole data collection was automated by *ZOO* system, including sample change by a robot^27^. Serial Synchrotron Rotation Crystallography (SS-ROX) method, which was developed to collect diffraction data of microcrystals efficiently, was employed^26,41^. A microbeam of 1.2 μm (vertical) × 1.0 μm (horizontal) was used. The datasets were collected using a helical rotation of 0.25° and translation of 1 μm per frame with a frame rate of 58.824 Hz (~ 2 × 10^10^ photons/frame). Indexing was performed using *CrystFEL* version 0.6.3^42^ with *Dirax*^43^ and *Mosflm*^44^. The number of indexed images for **PhC_CF**_**20°C/6h**_, **PhC_CF**_**20°C/12h**_, **PhC_CF**_**20°C/24h**_, **PhC_CF**_**15°C/24h**_, and **PhC_CF**_**25°C/24h**_ are 1531, 8754, 12,846, 3834, and 12,376, respectively. Integrated intensities were merged by process_hkl in the *CrystFEL* suite. The datasets were not obtained for **PhC_CF**_**20°C/2h**_, **PhC_CF**_**20°C/4h**_, **PhC_CF**_**4°C/24h**_, and **PhC_CF**_**10°C/4h**_ due to fewer indexed images (1, 235, 0, and 258, respectively) (Table S1). The structure of **PhC_CF**_**20°C/24h**_ was solved by rigid-body refinement with *phenix.refine*^45^ using the previously determined structure (PDB ID: 5GQM). The refinement of the protein structure was performed using *phenix.refine* and *REFMAC5* in the *CCP4* suite^45,46^. Rebuilding was completed using *COOT*^47^ based on sigma-A weighted 2|*F*_o_*|* − |*F*_c_*|* and |*F*_o_*|* − |*F*_c_| electron density maps. The models were subjected to quality analysis during the various refinement stages with omit maps and *RAMPAGE*^48^.

### Cell-free synthesis and crystallization of CipA

Expression and crystallization of CipA were performed with dialysis method using a WEPRO7240 Expression Kit (CellFree Sciences). Transcription reaction was performed with the same as PhC_CF. Translation reactions were carried out using the dialysis method. The reaction mixture containing 20 μL of WEPRO7240 wheat germ extract including 40 mg/mL creatine kinase, 20 μL of prepared mRNA, and 40 μL of SUB-AMIX SGC solution was dialyzed against 2.5 mL SUB-AMIX SGC solution at 20 °C for 72 h. As for crystallization of CipA with additives, the reaction mixture containing 16 μL of WEPRO7240 wheat germ extract including 40 mg/mL creatine kinase, 16 μL of prepared mRNA, 16 μL additive of appropriate concentration, and 32 μL of SUB-AMIX SGC solution was dialyzed against 2.5 mL SUB-AMIX SGC solution including additives at 20 °C for 72 h. The concentrations of additives in reaction mixture and dialysis solution were 3 v/v%, 3 v/v%, 1 v/v%, 1 w/v %, 2 w/v %, and 1 v/v % for EtOH, Dioxane, PEG400, PEG3350, PEG8000, Dextran, and TEG, respectively. The crystals were collected by centrifuge and washed PBS several times.

### X-ray crystal structure analysis of the CipACs

Before the data collection, the crystals were immersed in PBS buffer containing 50% ethylene glycol and then loaded onto the MicroLoops (Mitegen), followed by frozen in liquid nitrogen. The diffraction data of CipACs were collected at 100 K using the beamline of BL32XU at SPring-8 with an X-ray wavelength of 1.00 Å. The crystal positions in a cryo-loop were identified by a low-dose raster scan. The complete datasets were obtained by merging multiple small-wedge (10° each) datasets collected from single crystals. Collected datasets were automatically processed and merged by *KAMO*^49^. Each dataset was indexed and integrated using *XDS*^50^. The datasets were subjected to hierarchical clustering by a pairwise correlation coefficient of intensities. The datasets in each cluster were scaled and merged using outlier rejections implemented in *KAMO*. The groups with the highest CC_1/2_ were chosen for downstream analyses. Twinning was analyzed with *phenix.xtriage*^51^. The structure was solved by molecular replacement with *Phaser-MR*^51^ of *PHENIX* suite using the predicted structure by AlphaFold2^31^. Rebuilding was completed using *COOT*^47^ based on sigma-A weighted 2|*F*_o_*|* − |*F*_c_*|* and |*F*_o_*|* − |*F*_c_| electron density maps. The models were subjected to quality analysis during the various refinement stages with omit maps and *RAMPAGE*^48^.

## Supplementary Information

Supplementary Information.

## Data Availability

Atomic coordinate of **PhC_CF**_**20°C/24h**_, **PhC_CF**_**20mL_20°C/24h**_ and **CipAC** crystallized with 3% 1, 4-dioxane have been deposited in the Protein Data Bank under the code of 7XHR, 7XWS and 7XHS, respectively. All other data or sources are available from the corresponding authors on reasonable request.
