# Supplementary material for: Cell-free protein crystallization for nanocrystal structure determination
Source: Sci Rep. 2022 Oct 3;12:16031. doi: 10.1038/s41598-022-19681-9 (PMC9530169; doi:10.1038/s41598-022-19681-9)
Supplement: Supplementary file 1 — Supplementary Information. [file 41598_2022_19681_MOESM1_ESM.pdf]

Supplementary information

Cell-free Protein Crystallization for Nanocrystal Structure Determination

Satoshi Abe,[*,1] Junko Tanaka,[1] Mariko Kojma,[1] Shuji Kanamaru,[1] Kunio Hirata,[2] Keitaro Yamashita,[2,4] Ayako Kobayashi,[1] and Takafumi Ueno[*,1,3]

[1]School of Life Science and Technology, Tokyo Institute of Technology, Nagatsuta-cho 4259, Midori-ku, Yokohama 226-8501, Japan

[2]SR Life Science Instrumentation Unit, RIKEN/SPring-8 Center, 1-1-1, Kouto, Sayo-cho, Sayo-gun, Hyogo 679-5148, Japan

[3]International Research Frontiers Initiative (IRFI), Tokyo Institute of Technology, Nagatsuta-cho 4259, Midori-ku, Yokohama 226-8501, Japan

[4]Present address: MRC Laboratory of Molecular Biology, Francis Crick Avenue, Cambridge CB2 0QH, United Kingdom

Correspondence and requests for materials should be addressed to S.A and T.U. (email: saabe@bio.titech.ac.jp, tueno@bio.titech.ac.jp).

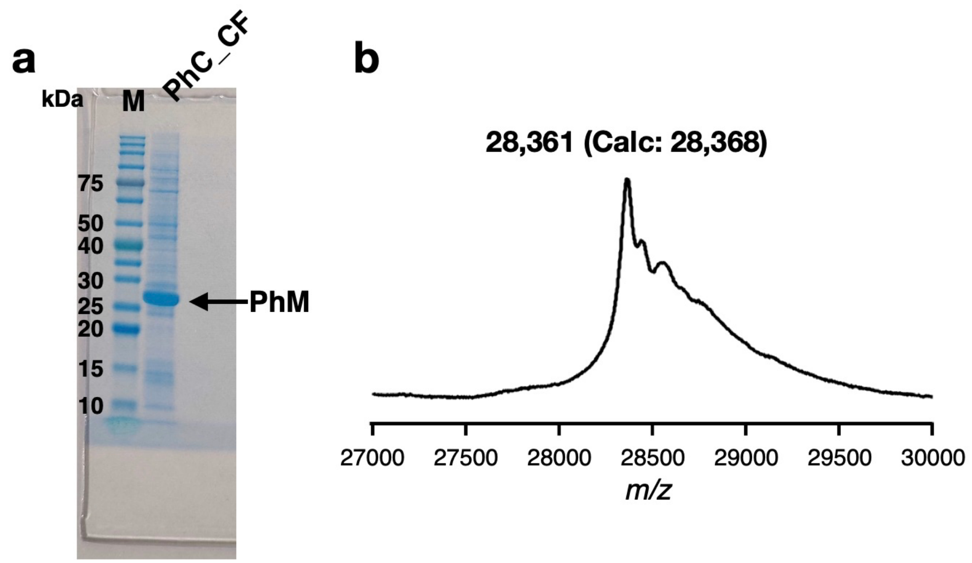

Supplementary Figure 1. (a) Coomassie-stained SDS-PAGE gel and (b) MALDI TOF-MS of purified **PhC_CF**.

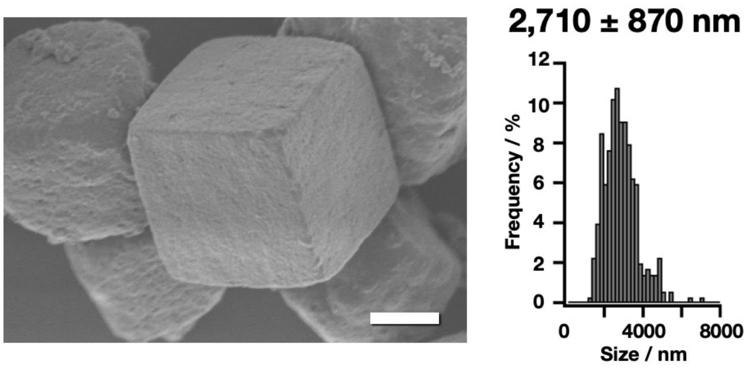

Supplementary Figure 2. SEM image and size histogram of purified PhC_IC. Scale bar = 1 μm.

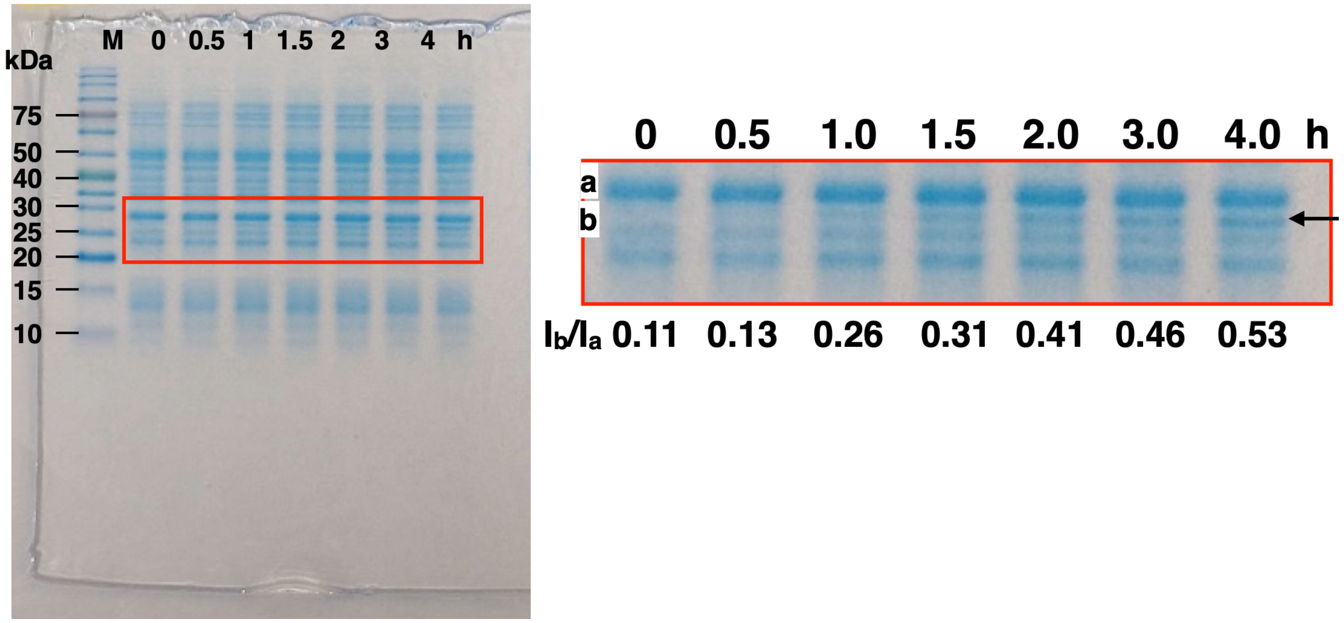

Supplementary Figure 3. Coomassie Blue stained SDS-PAGE gel of time-dependent expression of PhM at 20 °C. Uncropped and enlarged images of the red-frame area. The intensities of the bands are measured with Image J. The ratio (Ib/Ia) of the intensity of band b corresponding to PhM to that of band a is shown. Arrow indicates PhM.

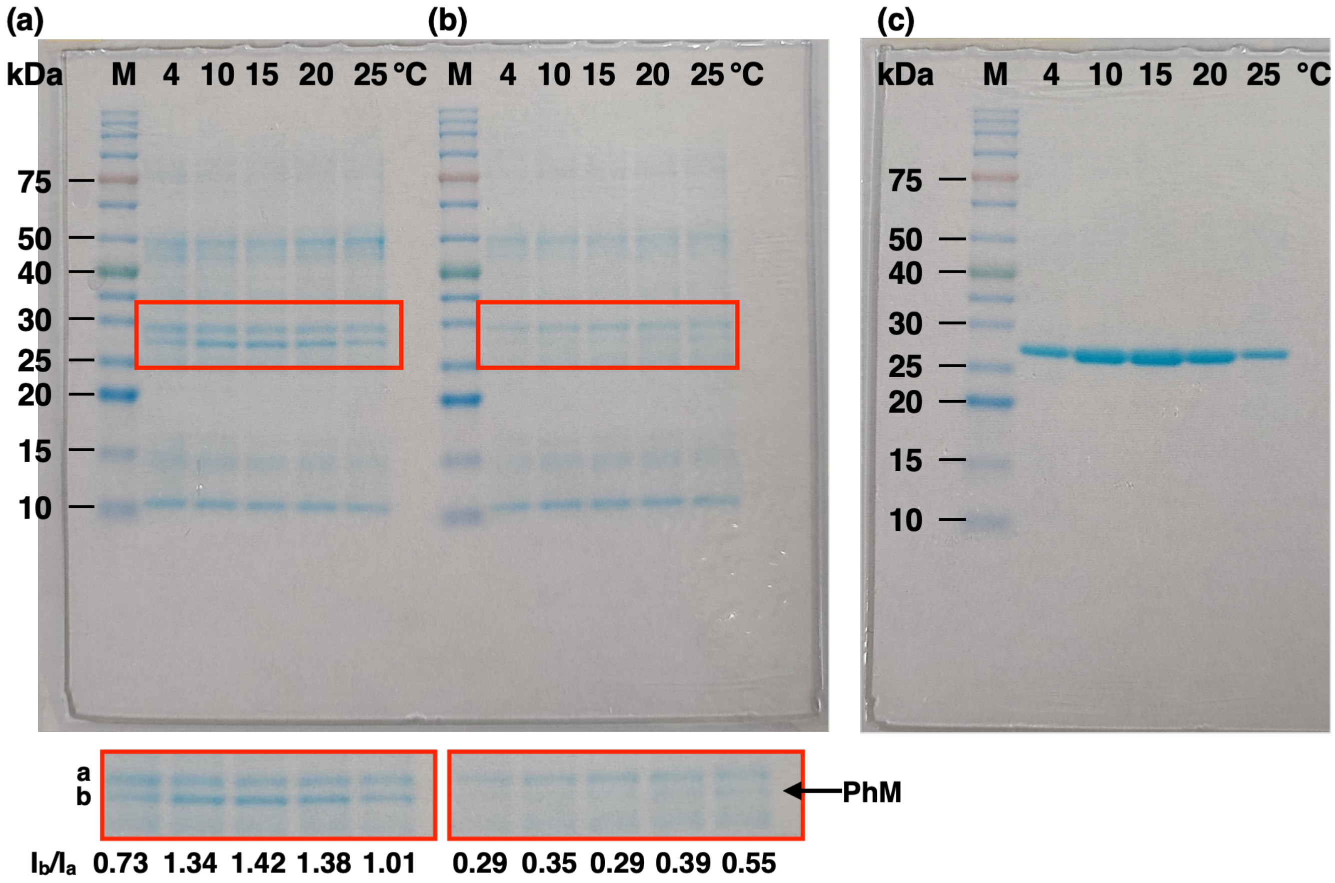

Percentage of crystallized PhM in synthesized PhM

Temperature / °C	4	10	15	20	25
Percentage / %	60	74	80	72	46

Supplementary Figure 4. Coomassie Blue stained SDS-PAGE gel of temperature-dependent expression and crystallization of PhM for 24 h. SDS-PAGE images of (a) reaction solution, (b) supernatant solution after purification by centrifugation, and (c) purified crystals. Uncropped and enlarged images of the red-frame area. Arrow indicates PhM. The intensities of the bands are measured with Image J. The ratio (Ib/Ia) of the intensity of band b corresponding to PhM to that of band a is shown. The percentage of crystallized PhM in total PhM was calculated based on the intensity at each temperature.

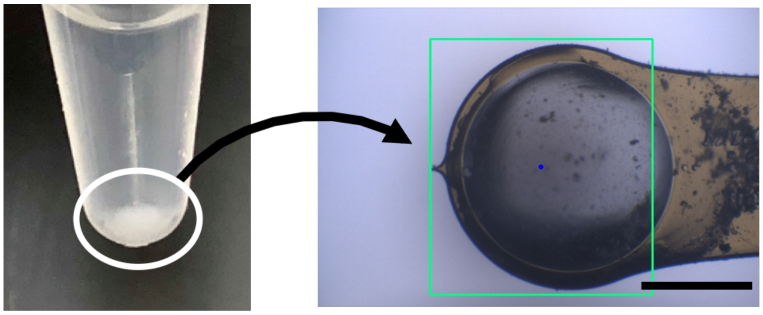

Supplementary Figure 5. Photograph of the tube after CFPC of **PhC_CF** and the MicroLoops used for diffraction experiment at the BL32XU of SPrin-8. Scale bar = 500 μm.

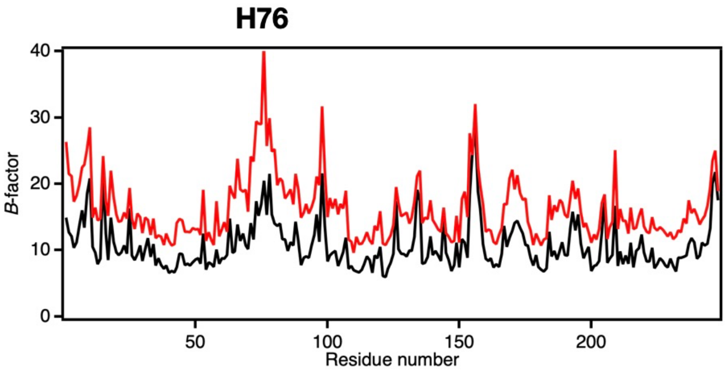

Supplementary Figure 6. Average *B*-factor values per residue of all atoms in **PhC_CF$_{\mathbf{20°C/24h}}$** (red line) and PhC_IC (black line).

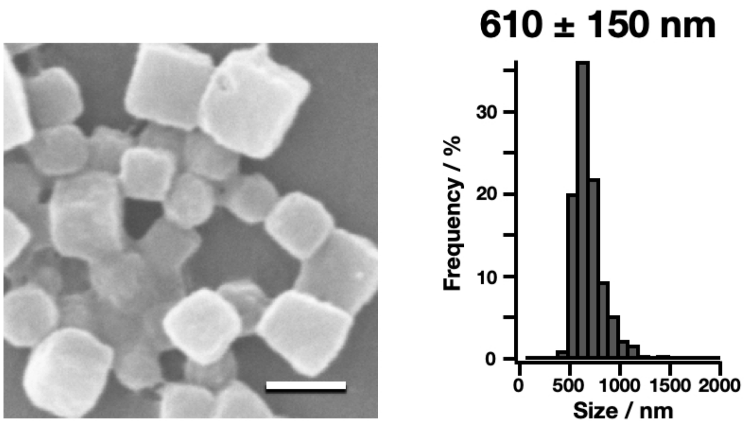

Supplementary Figure 7. SEM image and size histogram of **PhC_CF** produced by dialysis with 20 μL reaction scale. Scale bar = 1 μm

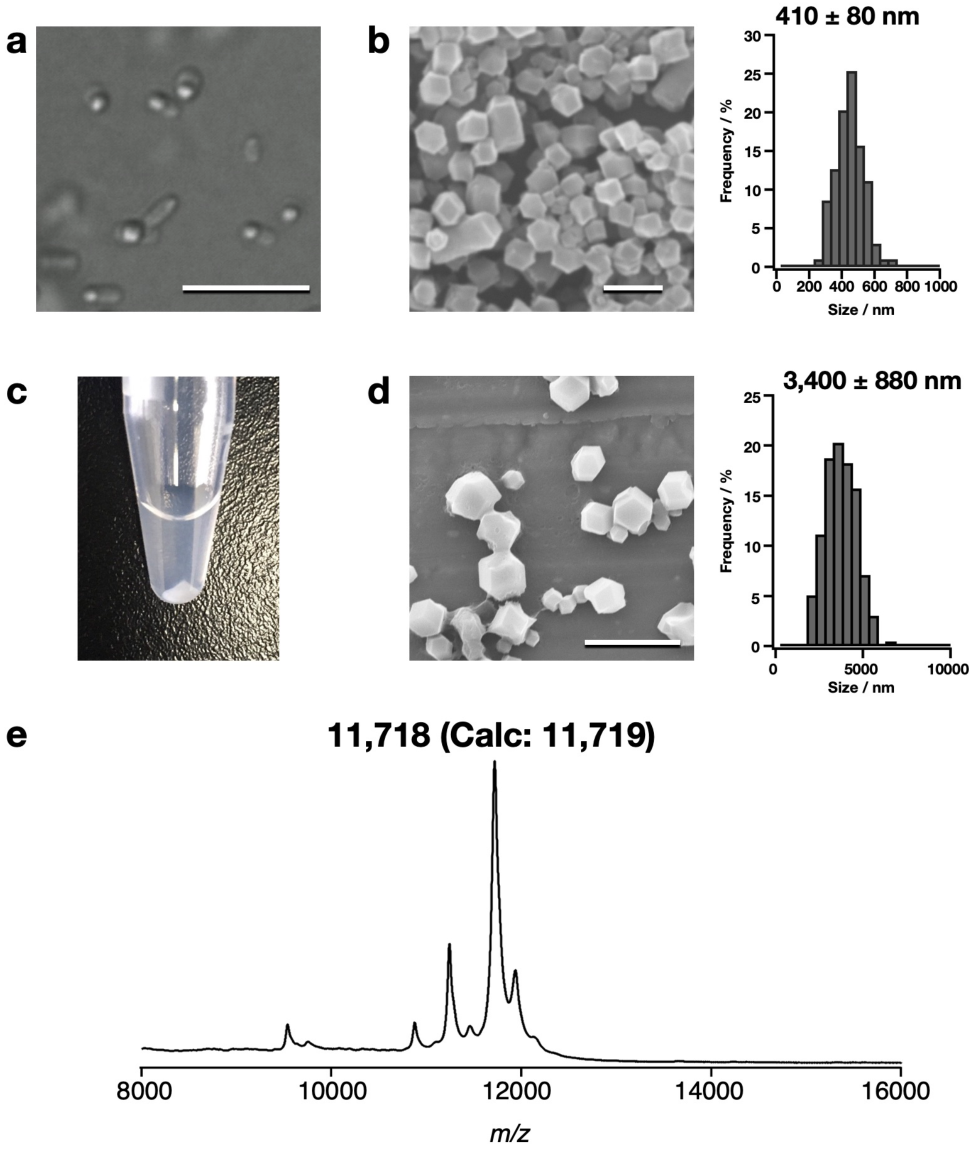

Supplementary Figure 8. (a) *E.coli* produced CipAC. Scale bar = 10 μm. (b) SEM image and size histogram of purified CipAC_EC. Scale bar = 1 μm. (c) Photograph of the tube after CFPC of CipA. (d) SEM image and size histograms of purified **CipAC_CF**. Scale bar = 10 μm. (e) MALDI TOF-MS of purified **CipAC_CF**.

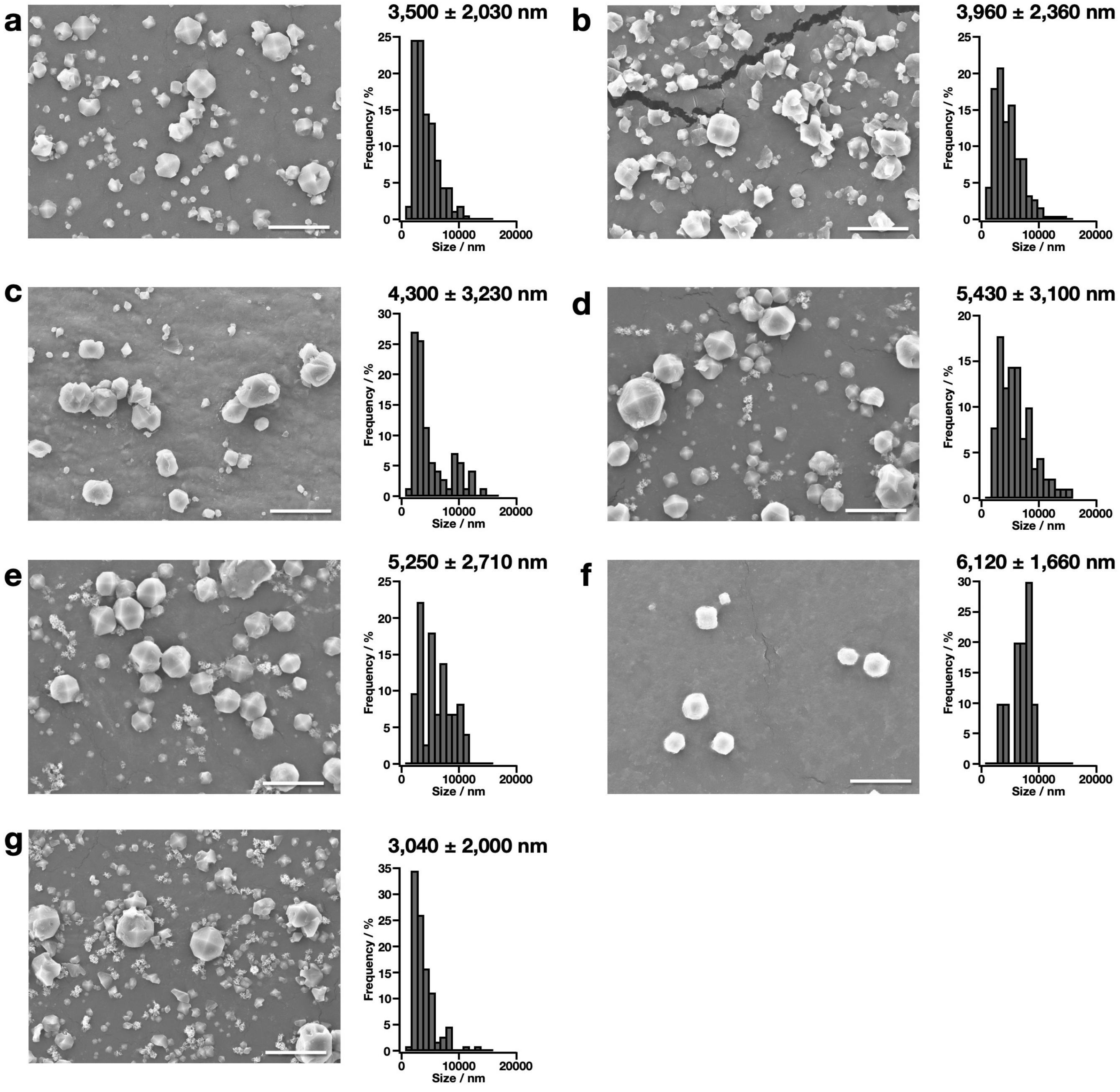

Supplementary Figure 9. SEM images of purified **CipAC_CFs** with additives. (a) 3 v/v % EtOH, (b) 3 v/v% Dioxane, (c) 1 v/v% PEG400, (d) 1 w/v % PEG3350, (e) 1 w/v % PEG8000, (f) 2 w/v % Dextran, (g) 1 v/v% TEG. Scale bars = 20 μm

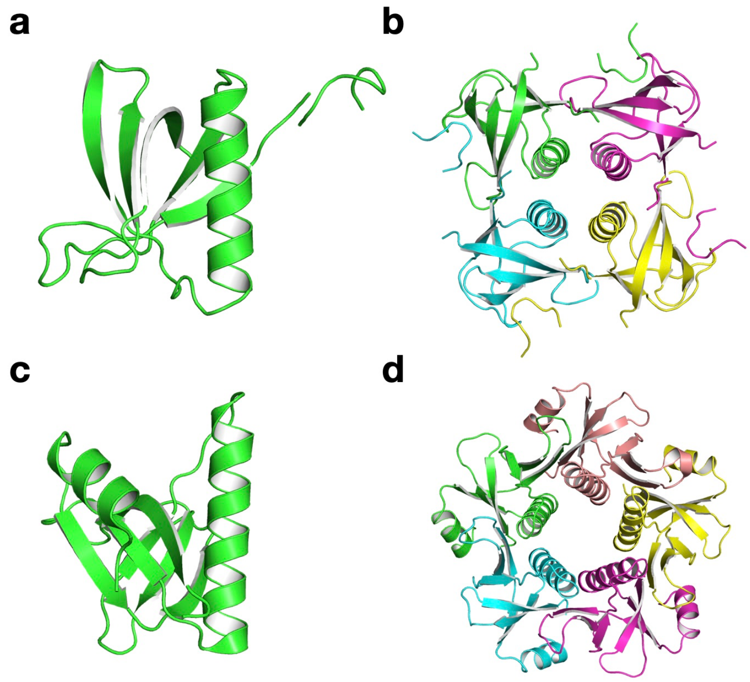

Supplementary Figure 10. Structure comparison of (a and b) CipA and (c and d) heat-labile enterotoxin type IIB (PDB ID: 1QB5).
